# Land use changes and its driving forces in hilly ecological restoration area based on gis and rs of northern china

**DOI:** 10.1038/srep11038

**Published:** 2015-06-05

**Authors:** Peng Gao, Xiang Niu, Bing Wang, Yunlong Zheng

**Affiliations:** 1 Shandong Agricultural University, College of Forestry; Taishan Mountain Forest Ecosystem Research Station, Tai’an, Shandong 271018, P.R. China; 2 Research Institute of Forest Ecology, Environment and Protection, Chinese Academy of Forestry, Beijing 100091, China

## Abstract

Land use change is one of the important aspects of the regional ecological restoration research. With remote sensing (RS) image in 2003, 2007 and 2012, using geographic information system (GIS) technologies, the land use pattern changes in Yimeng Mountain ecological restoration area in China and its driving force factors were studied. Results showed that: (1) Cultivated land constituted the largest area during 10 years, and followed by forest land and grass land; cultivated land and unused land were reduced by 28.43% and 44.32%, whereas forest land, water area and land for water facilities and others were increased. (2) During 2003–2007, forest land change showed the largest, followed by unused land and grass land; however, during 2008–2012, water area and land for water facilities change showed the largest, followed by grass land and unused land. (3) Land use degree was above the average level, it was in the developing period during 2003–2007 and in the degenerating period during 2008–2012. (4) Ecological Restoration Projects can greatly change the micro topography, increase vegetation coverage, and then induce significant changes in the land use distribution, which were the main driving force factors of the land use pattern change in the ecological restoration area.

Land use/cover change (LUCC) is one of the main causes of global change, and it is the issues most closely related to natural and human processes, affecting the sustainable development of cities, societies and people’s daily lives^1^,^2^. Facing the current increasingly severe problems of the population-resource-environment balance, the research on LUCC has become the frontier and a hot issue in global change^3^,^4^. In 2002, LUCC research has entered into the phase of Land Project, and IGBP(International Geosphere-Biosphere Program) formulated the research emphasis and related scientific problems of Land Project^5^. The research contents have extended from the global climate change effects to the LUCC process of different scales, the driving mechanism and its influences on environmental resources^6^,^7^,^8^.

Fu *et al.* thought that the driving forces of LUCC were climate change and human activities, and so its driving force index should include two types of bio-physical and socio-economical types^9^. Bakkera *et al.* studied the abandoned farmland problem of Lesvos island in Greek, and found the soil erosion as a driver of land-use change^10^. Zhang *et al.* analyzed the quantities, inner structure, types and spatial distribution features of LUCC on the Loess Plateau of east Gansu in the last fifteen years of the 20th century and discovered that the main driving forces of LUCC on the Loess Plateau of east Gansu were natural factors, economic development, population pressure, the adjustment of macro policies, and so on^11^. By selecting the elevation and slope as the index of land use change driving forces, Ye *et al.* found that their relationship with land use change was obvious^12^. Zou *et al.* studied land use change dynamics spatial patterns in an ecotone between agriculture and animal husbandry in China and analyzed its driving force; he found that the Tibetan Plateau uplift was the important driving force of climate change in the northern hemisphere of the late Cenozoic and that it had a significant effect on the eastern grassland changes of China^13^. Especially in recent years, it has become the latest trend to research the spatial change rules of LUCC and its driving force factors in different periods of time using the Erdas Imagine remote sensing (RS) and geographic information system (GIS) technologies^14^,^15^. So, some significant achievements of researching the driving mechanisms based on the biophysical factors and socio-economic factors have been reported^16^,^17^.

In 1999, the Ecological Restoration Projects was initiated in China by restoring forest vegetation on wasteland and cropland, one of the world’s most ambitious conservation set-aside programs, and the nation’s largest ecological restoration project since the 1970 s^18^,^19^. The ecological restoration measures mainly include the cropland afforestation, wasteland afforestation, facilitate afforestation, soil conservation tillage, construction of a terrace, comprehensive improvement on gully dam system, etc. The project has ambition to increase area of forest and grassland to 29.8 × 10^6^ ha by 2013 (refer to the Annual Reports on the Development of Chinese Forestry (1999–2013) edited by State Forestry Administration, PR China). It is very important to research the land use changes and driving forces of the Ecological Restoration Projects, which can provide a scientific base for the regulation of ecological restoration measures and its beneficial quantitative evaluation. At present, there is a large amount of research on the technologies of the Ecological Restoration Projects and their benefits^20^,^21^,^22^. However, few studies have characterized the dynamic changes of land use patterns of the Ecological Restoration Projects based on RS and GIS in the study area. Therefore, it is difficult to accurately answer the questions of ecological restoration mechanisms. Selecting the ecological restoration area of Yimeng Mountain in Shandong Province, a typical hilly area in the northern part of China, the dynamic changes of land use patterns of the hilly ecological restoration area were quantitatively analyzed using Landsat TM RS images from 2003, 2007 and 2012, using RS and GIS technologies, and its driving force factors were discussed in the study area.

## Results

### Change features of land use structure

Table 1 showed that the most widespread land use type during the past 10 years in the study area was cultivated land, accounting for 38.80%–54.21% of the total land use, followed by forest land and grass land, accounting for 16.97%–31.17% and 10.58%–15.35% of the total land use, respectively. The proportion of cultivated land and unused land with respect to the total area were reduced by 28.43% and 44.32%, respectively, whereas the proportions of forest land, grass land, urban village and mining traffic land, water area and land for water facilities were increased by 83.68%, 10.75%, 21.07% and 162.74%, respectively.

### Land use change rate

The land use dynamic degree (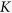) of all land use types in the study area were calculated using formula (1) (Table 1). During 2003–2007, the 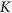 value of forest land was the largest, accounting for 12.42% of the change in total land use, and it indicated that the change amplitude of forest land was the biggest, followed by those of unused land and grass land, accounting for – 5.22% and – 4.73% of the change in total land use, respectively; water areas and land for water facilities, urban village and mining traffic land and cultivated land changed relatively less, accounting for 3.68%, 2.36% and – 2.01% of the change in total land use, respectively. During 2008–2012, the 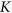 value of water areas and land for water facilities was the highest, accounting for 24.82% of the change in total land use, and its change amplitude was the biggest, followed by grass land, unused land and cultivated land, accounting for 9.01%, – 4.92% and – 4.09% of the change in total land use, respectively; the change in forest land, urban village and mining traffic land was relatively smaller, accounting for 2.66% and 1.66% of the change in total land use, respectively.

### Land use change degree

The land use degree comprehensive index (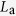) in the study was calculated using formula (2) and (3) (Table 2). 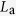 is a continuous function whose value interval is between [100, 400], which reflects the land use degree. The larger 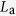, the higher of the land use degree. Table 2 showed that the values of 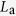 in the study area in 2003, 2007 and 2012 were 240.47, 240.89 and 235.06, respectively, which indicated that the land use degree and development intensity in the study area was above the average level. From Table 2, 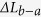 was positive (+0.42) during 2003–2007 in the study area, which indicated that the land utilization was in the improving and developing stage; during 2008–2012, 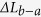 was negative (– 5.83), which indicated that the land utilization was in the adjusting stage in the study area.

### Land use type transformation Feature

**Figure 1:**
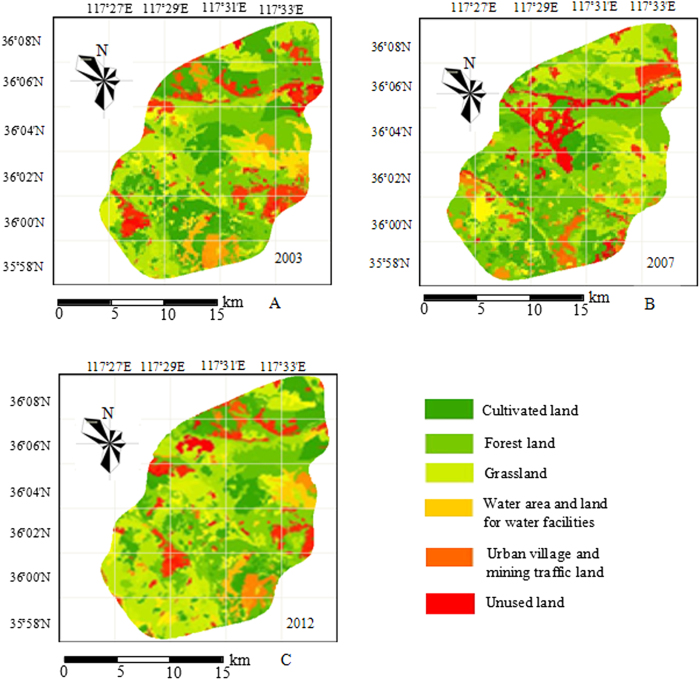


From Table 3 and Fig. 1A,B, 70.76% of the mapping unit of land use types was unchanged during 2003–2007. The land use transfer matrix during this period happened mainly between the cultivated land, forest land and grassland; 66.5 km^2^ and 22.4 km^2^ of the cultivated land transferred to forest land and grassland, accounting for 73.77% and 24.84% of the total transfer area, respectively. Moreover, the unused land also transferred obviously, 5.3 km^2^ and 4.4 km^2^ of which have transferred into urban village and mining traffic land and forest land, respectively, accounting for 28.00% and 23.73% of the total, respectively. Water areas and land for water facilities as well as urban village and mining traffic land grew relatively less; their main sources were unused land and small amounts of cultivated land.

Table 4 and Fig. 1B,C showed that 71.47% of the mapping unit of land use types was unchanged during 2008−2012. Similarly, during this period, the land use transfer matrix occurred mainly between the cultivated land, forest land and grassland; 66.6 km^2^ and 37.1 km^2^ of the cultivated land transferred into forest land and grassland, respectively, accounting for 59.16% and 32.99% of the total, respectively. The total area of the water areas and land for water facilities increased by 21.3 km^2^ and mainly came from unused land and some cultivated land. The area of unused land decreased by 12.6 km^2^, which mainly transferred to forest land and water areas and land for water facilities. The area of urban village and mining traffic land increased by 2.0 km^2^, which mainly comes from unused land and some low-yield cultivated land.

### Analysis of driving force factors of land use change

The calculation results of contribution rate of each principal component showed that the cumulative contribution rate of the first(Y_1_) and second (Y_2_) principal component factors has exceeded 85.064%(Table 5), so it showed the explain ability of the 9 driving force factors reached 85.064%, and meet the requirements of analysis. And the load matrix of principal components was obtained by the maximum variance method (Table 6).The first (Y_1_) and second (Y_2_) principal component factor expressions are as follows.


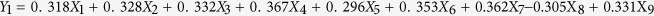



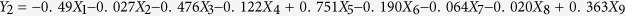


Table 6 also showed that the driving force factors of the closely related to the first (Y_1_) principal component factor were X_4_, X_7_, X_6_, X_3_ and X_9_, and their correlation coefficients were all above 0.884, which represented the degree of the land development, planting, animal husbandry and forestry development in Yimeng Mountain ecological restoration area. In addition, the correlation coefficients of X_2_ and X_1_ were also above 0.849, so natural factors, such as terrain slope(X_2_) and annual precipitation(X_1_) were also the important influencing factors of land use change.

## Discussions

### Driving force feature of the land use chang**e**

Land use change is a direct manifestation of human effects on the natural environment, whose development is mainly affected by natural and human factors^23^. Natural factors are fundamental to the land use distribution of the ecological environment, which include altitude, landform, gradient, slope direction, soil, vegetation, etc.^24^, and human factors which include population, economy, system policy, technical measures, etc.^25^,^26^. In our study, the results indicated that the social and economic development factors, such as land reclamation rate, per capita amount of stock raising, per capita forest and grass area, per capita grain output and population density, were the main driving force factors in the land use change of Yimeng Mountain ecological restoration area(Table 5,Table 6), and these driving factors represented the degree of land development, planting, animal husbandry and forestry development in the study area. Moreover, the natural factors, such as terrain slope and annual precipitation, also were the important influencing factors on the land use change. These results were similar to the results found by Wu *et al.*^27^.

### Ecological Restoration Project significantly influence on the land use change

The above driving force factors(X_2_, X_4_, X_7_, X_6_, X_3_, X_9_) were closely associated with the ecological restoration measures(such as the cropland afforestation, wasteland afforestation, facilitate afforestation, soil conservation tillage, construction of a terrace, comprehensive improvement on gully dam system, etc.) in Yimeng Mountain area. In 2003 (before the implementation of ecological restoration measures), cultivated land accounted for the largest proportion of the land use, accounting for 54.21% of the total, and unused land accounted for 9.86% of the total use, which together account for 64.07% of the land use. During the ten years from 2003 to 2012, the implementation of the Ecological Restoration Project brought a significant decrease in the cultivated and unused land, the proportion of which decreased by 28.43% and 44.32%, respectively, and the transformation from cultivated and unused land into ecological forest land, economic forest land and grassland was the major pattern of land use change. Therefore, the distribution and change of the cultivated and unused land mainly influenced the terrain distribution pattern of land use all over the ecological restoration area.

According to the data of Landsat TM RS images from 2003, 2007 and 2012, and the other research results^28^, The cultivated land in the study area is mainly sloped cropland of the hilly area, and the slope is relatively low on the whole. During the ten years since the implementation of the Ecological Restoration Project, the construction of a terrace and the increase in yield per unit of sloped cropland have led to more sloped cropland (cultivated land) transferring to forest land. Because the reduced sloped cropland was mainly distributed in the lower part of the sloped surface, the average slope of the cultivated land was decreased significantly and the average altitude increased in the study area.

During the ten year period, the Ecological Restoration Project, which controlled an area of 75 km^2^ and an open forest planting area of 80.5 km^2^, successively developed an area of economic forest grass of 45 km^2^ under high standard land preparation, which resulted in the proportion of the forest land and grass land accounting for the total area increasing by 83.68% and 10.75%, respectively (Table 1). Moreover, the construction of the channel check dam and reservoir in the study area resulted in the proportion of the water area and land for water facilities increasing by 162.21% (Table 1). Therefore, the implementation of the Ecological Restoration Project greatly changed the landform, slope and vegetation coverage of the study area, which played an important influence on the land use change of Yimeng Mountain ecological restoration area.

## Conclusions

(1) The land use pattern in the ecological restoration area in Yimeng Mountain has changed significantly during the 10 years of this research. The cultivated land maintained the largest area, followed by forest land and grass land. Moreover, the proportion of the total area that was cultivated land and unused land decreased by 28.43% and 44.32%, respectively, whereas forest land, grass land, urban village and mining traffic land, and water area and land for water facilities increased by 83.68%, 10.75%, 21.07% and 162.74%, respectively.

(2) The analysis of the land use dynamic degree showed that the extent of forest land use change was the largest during 2003–2007, accounting for 12.42% of the change in land use, followed by unused land and grass land. However, the change of water areas and land for water facilities was the largest during 2008–2012, accounting for 24.82% of the change in land use, followed by grass land, unused land and cultivated land.

(3) The analysis of the land use degree comprehensive index indicated that the land use and development degree in the study area was higher than the average level and that it was in the developing period during 2003–2007 and in the degenerating period during 2008–2012 and that the transformation of land use types mainly occurred in cultivated land, forest land and grass land.

(4) The effects of human activities on the spatial distribution of land use are the main driving force factors of the land use pattern change, which can lead to great changes over a short period, especially the implementation of Ecological Restoration Projects, which can greatly change the micro topography, reduce the surface slope, increase the vegetation coverage, and then induce significant changes in the spatial distribution of land use in the ecological restoration area of Yimeng Mountain in the northern part of China.

## Materials and methods

The project area setup, observation indicators and test methods were all based on the Specifications for Assessment of Forest Ecosystem Services in China (LY/T 1721–2008), Indicators System for Long-term Observation of Forestry Ecosystems (LY/T 1606–2003) and Observation Methodology for Long-term Forest Ecosystem Research (LY/T 1952–2011)^29^.

### Study area and environmental conditions

**Figure 2:**
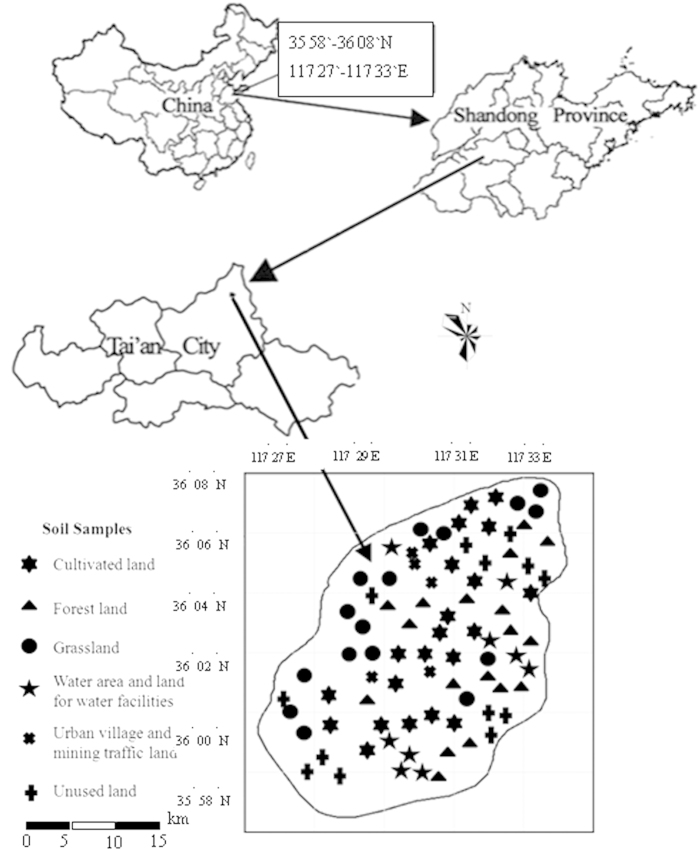


The experiment was conducted in Tai’an Xintai City and Shandong Province (35°58′-36°08′N,117°27′-117°33′E), located in Yimeng Mountain of south-central Shandong Province in China (Fig. 2), where the elevation ranges from 310 m to 413 m. This area has a typical monsoon climate and is located in a warm temperate zone with distinct seasonal changes. The mean annual precipitation is 798.4 mm, and nearly 70% of the annual precipitation falls between June and September. The average annual evaporation in this region is 1942.6 mm, and the mean annual temperature is approximately 12.0 °C. The soil type in this study is referred to as Brown soil and is similar to the American soil classification of Eutrochrepts; the average soil layer thickness is 20 cm; the soil pH is 6.5–6.9, and it shows higher soil and water loss. So improving the ecological environment management is necessary in the study area. The ecological restoration program of Yimeng Mountain was implemented in 2003, and divided into two parts, each period of five years, namely, 2003–2007 and 2008–2012. According to the floristic-vegetational analysis results^30^, the vegetation types in the study area belong to the coniferous forests and deciduous broad-leaved forests in the warm temperate zone and to the north China flora of China. Moreover, the coniferous forests include *Platycladus orientalis* (L.) and *Pinus thunbergii* Parl.; the deciduous broad-leaved forests include *Cotinus coggygria* Scop., *Robinia pseudoacacia* Linn., *Prunus Armeniaca* Mill., *Julans regia* Linn., etc.; the shrubs include *Vitex negundo* Linn. var. negundo and *Ziziphus jujuba* var.spinosa Hu, etc.; the species in the waste grassland are *Zoysia japonica* Steud., *Rubia manjith* Roxb. ex Flem., *Themeda japonica* Tanaka and *Setaria viridis (*Linn.) Beauv., etc.^28^.

### Date source

Based on the RS software (Erdas Imagine 8.7) and GIS (ArcGIS 9.3) technology, we have processed LANDSAT TM remote sensing images of the three typical periods, in 2003, 2007 and 2012 (Namely: on June 25, 2003; on June 9, 2007; on June 21, 2012. and multispectral image resolution is 30 m, the path/row is 122 /36). In order to reduce the error of image processing, it needed to geometric correction, image sharpening and cloud removal, and drew the plaques to carry out human-computer interaction translations according to the images of hue, saturation, shape, shadow, texture, position, and size. Then, we made a comprehensive analysis and correction on the translation results using the topographic map, a geological map, a soil map, the present land utilization data and a combined GPS survey of the location. Moreover, according to the national standard of *the classification of the present land use situation* (GB/T 21010–2007) and the actual situation in the study area, the land use in the study area was divided into six land use types, namely cultivated land, forest land, grass land, water area and land for water facilities, urban village and mining traffic land and unused land.

### Methods of the research

(1) Land use dynamic degree (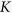): To quantitatively describe the range and speed of LUCC, the land use dynamic degree was introduced, the equation to calculate 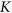 is as follows^31^,^32^.


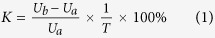


where 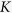 is the land use dynamic degree of a specific land use type, defined as percent land use change per year; 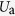 and 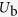 represent the area under a specific land use type per year, respectively; 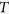 is time in years.

(2) Land use degree comprehensive index (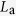): This index mainly reflects the impact of human factors in the land system; to quantitatively measure the intensive land use level, Zhuang *et al.* posited the classification principles, the classification values of land use degree classification index (Table 7), and the expression of 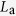
33.


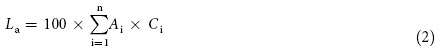


where 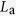 is the land use degree comprehensive index, its value interval is between [100, 400]; 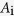 is the land use degree classification index of the i^th^ class; 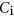 is the land use degree classification area percentage of the i^th^ class; and 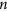 is the land use degree classification number.

By using the land use degree comprehensive index, we can obtain the land use degree change value; its expression is as follows:


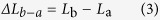


where 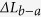 is the land use degree change value; 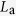 and 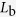 represent the land use degree comprehensive index of time a and b of the i^th^ class, respectively.

If 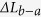 is positive, it would suggest the development period of the regional land use status; if 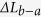 is negative, it would suggest the adjustment or recession period of the regional land use status.

(3) Land use transfer matrix: The dynamic transfer matrix can describe the reciprocal transformation between the land use types^34^, which can be used for the hilly ecological restoration area to simulate the process of land use and then form a land use dynamic change matrix table. Combined with the regional influence of Landsat TM data, we can fully explain a period of time during the exchange of various land use types.

(4) Analysis method of driving force factors of land use change: Based on the data of Landsat TM RS images from 2003, 2007 and 2012, and the relative data of statistical yearbook of history(2003–2012)^35^, the driving force of the land use pattern change in the ecological restoration area of Yimeng Mountain were further divided into the following 9 factors, namely: the annual precipitation(X_1_, mm), terrain slope(X_2_, °), population density(X_3_, person/km^2^), land reclamation rate(X_4_, %), per capita cultivated land area(X_5_, hm^2^/ per capita), per capita forest and grass area(X_6_, hm^2^/per capita), per capita amount of stock raising(X_7_, kg/per capita),per capita agricultural output value(X_8_, yuan/per capita), per capita grain output(X_9_, kg/per capita)(Table 8). And the principal component analysis method^36^ was applied to help understanding land use change mechanisms linked to these factors.

## Additional Information

**How to cite this article**: Gao, P. *et al.* Land Use Changes and Its Driving Forces in Hilly Ecological Restoration Area Based on GIS and RS of Northern China. *Sci. Rep.* **5**, 11038; doi: 10.1038/srep11038 (2015).

## Figures and Tables

**Table 1:** Land use dynamic degree of land use types of the ecological restoration area in Yimeng Mountain.

**Land use type**	**2003**		**2007**		**2012**		**Land use dynamic degree** ***K*****(%)**	
	**Area (km**^**2**^)	**Proportion (%)**	**Area (km**^**2**^)	**Proportion (%)**	**Area (km**^**2**^)	**Proportion (%)**	**2003–2007**	**2008–2012**
Cultivated land	378.4	54.21	340.5	48.77	270.9	38.80	−2.01*	−4.09
Forest land	118.5	16.97	192.0	27.50	217.6	31.17	+12.42	+2.66
Grass land	96.8	13.86	73.9	10.58	107.2	15.35	−4.73	+9.01
water area and land for water facilities	14.8	2.12	17.6	2.51	38.9	5.57	+3.68	+24.82
Urban village and mining traffic land	20.9	2.99	23.3	3.34	25.3	3.62	+2.36	+1.66
Unused land	68.8	9.86	50.9	5.49	38.3	5.49	−5.22	−4.92

^*^“+” indicates the increase; “−” indicates the decrease.

**Table 2:** Land use degree comprehensive index of the ecological restoration area in Yimeng Mountain

**Study area**	**Land use degree comprehensive index** 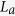			**Land use degree change value** 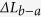		
Yimeng Mountain	2003	2007	2012	2003–2007	2008–2012	2003–2012
ecological restoration area	240.47	240.89	235.06	+0.42	−5.83	−5.41

**Table 3:** Land use transfer matrix of the ecological restoration area in Yimeng Mountain during 2003–2007 (km^2^).

**Dynamic transfer matrix**	**2003**							**2007 Total**
		1*	**2**	**3**	**4**	**5**	**6**	
2007	1	288.2	21.8	24.6	1.2	2.0	2.5	340.5
	2	66.5	90.0	27.7	1.1	2.3	4.4	192.0
	3	22.4	4.3	43.3	0.1	1.1	2.7	73.9
	4	0.1	1.1	0.6	12.0	0.1	3.8	17.6
	5	0.9	1.0	0.4	0.3	15.4	5.3	23.3
	6	0.3	0.3	0.2	0.1	0.0	50.1	50.9
2003 Total		378.4	118.5	96.8	14.8	20.9	68.8	698.2

^*^1 Cultivated land, 2 Forest land, 3 grassland, 4 Water area and land for water facilities, 5 Urban village and mining traffic land, 6 Unused land.

**Table 4:** Land use transfer matrix of the ecological restoration area in Yimeng Mountain during 2008–2012 (km^2^).

**Dynamic transfer matrix**	**2008**							**2012 Total**
		1*	**2**	**3**	**4**	**5**	**6**	
2012	1	228.0	34.4	6.7	1.0	0.6	0.1	270.9
	2	66.6	140.3	6.0	0.6	0.3	3.8	217.6
	3	37.1	10.3	56.9	0.4	0.4	2.2	107.2
	4	7.2	5.9	3.7	15.0	0.4	6.6	38.9
	5	1.5	0.8	0.3	0.4	21.4	0.9	25.3
	6	0.1	0.3	0.3	0.2	0.2	37.3	38.3
2008 Total		340.5	192.0	73.9	17.6	23.3	50.9	698.2

^*^1 Cultivated land, 2 Forest land, 3 grassland, 4 Water area and land for water facilities, 5 Urban village and mining traffic land, 6 Unused land.

**Table 5:** Characteristic value and contribution rates of each principal component.

**Principal components**	**Driving forcefactors**	**Characteristic value**	**Contribution rate (%)**	**Cumulative contribution rate (%)**
Y_1_	X_1_*	7.112	79.017	79.017
Y_2_	X_2_	0.544	6.047	85.064
Y_3_	X_3_	0.462	5.137	90.201
Y_4_	X_4_	0.423	4.697	94.899
Y_5_	X_5_	0.201	2.229	97.128
Y_6_	X_6_	0.120	1.331	98.459
Y_7_	X_7_	0.082	0.911	99.370
Y_8_	X_8_	0.030	0.339	99.709
Y_9_	X_9_	0.026	0.291	100.000

^*^X1 annual precipitation, X_2_ terrain slop, X_3_ population density, X_4_ land reclamation rate, X_5_ per capita cultivated land area, X_6_ per capita forest and grass area, X_7_ per capita amount of stock raising, X_8_ per capita agricultural output value, X_9_ per capita grain output.

**Table 6:** Load matrix of principal components.

**Driving force factors**	**Principal component(Y**_**1**_)	**Principal component(Y**_**2**_)	**Principal component(Y**_**3**_)	**Principal component(Y**_**4**_)
X_1_*	0.849	−0.110	−0.137	0.435
X_2_	0.874	−0.020	0.391	−0.169
X_3_	0.885	−0.351	−0.026	−0.188
X_4_	0.980	−0.090	0.041	−0.070
X_5_	0.790	0.554	−0.007	−0.182
X_6_	0.941	−0.140	0.090	0.025
X_7_	0.966	−0.047	0.059	0.028
X_8_	−0.813	−0.014	0.523	0.197
X_9_	0.884	0.268	0.052	0.301

^*^X1 annual precipitation, X_2_ terrain slop, X_3_ population density, X_4_ land reclamation rate, X_5_ per capita cultivated land area, X_6_ per capita forest and grass area, X_7_ per capita amount of stock raising, X_8_ per capita agricultural output value, X_9_ per capita grain output.

**Table 7:** Classification values of land use degree classification index

**Land use classification**	**Uncultivated land level**	**Grass and water with ground level**	**Agriculture land level**	**Urban community land level**
Land use type	Unused (waste mountains)	Forest grassland, water area and land for water facilities	Cultivated land, garden land, artificial grass	Urban village and mining traffic land
Land use degree classification index: *A*_i_	1	2	3	4

**Table 8:** Driving force factors of the land use change of the ecological restoration area in Yimeng Mountain.

**Types and content of driving force factors**			**Units of driving force factors**	**Source of the data**
Natural factors	X_1_	annual precipitation	mm	Meteorological data of Bureau of meteorology of Xintai City
	X_2_	terrain slope	°	Data of Landsat TM RS images from 2003, 2007 and 2012
Social and economic development factors	X_3_	population density	person/km^2^	Data of statistical yearbook of history(2003–2012) and investigation
	X_4_	land reclamation rate	%	Data of statistical yearbook of history(2003–2012) and investigation
	X_5_	per capita cultivated land area	hm^2^/ per capita	Data of statistical yearbook of history(2003–2012) and investigation
	X_6_	per capita forest and grass area	hm^2^/per capita	Data of statistical yearbook of history(2003–2012) and investigation
	X_7_	per capita amount of stock raising	kg/per capita	Data of statistical yearbook of history(2003–2012) and investigation
	X_8_	per capita agricultural output value	yuan/per capita	Data of statistical yearbook of history(2003–2012) and investigation
	X_9_	per capita grain output	kg/per capita	Data of statistical yearbook of history(2003–2012) and investigation
Land use change index	Y	land use change area	hm^2^	Data of Landsat TM RS images from 2003, 2007 and 2012 and Data of statistical yearbook of history(2003–2012) and investigation
